# Estimating the total genome length of a metagenomic sample using k-mers

**DOI:** 10.1186/s12864-019-5467-x

**Published:** 2019-04-04

**Authors:** Kui Hua, Xuegong Zhang

**Affiliations:** 1 MOE Key Laboratory of Bioinformatics Division and Center for Synthetic & System Biology, BNRIST, Beijing, 100084 China; 2 Department of Automation, Tsinghua University, Beijing, 100084 China; 3 School of Life Sciences, Tsinghua University, Beijing, 100084 China

**Keywords:** Metagenomics, Sequencing coverage, Distinct k-mers, Genome length

## Abstract

**Background:**

Metagenomic sequencing is a powerful technology for studying the mixture of microbes or the microbiomes on human and in the environment. One basic task of analyzing metagenomic data is to identify the component genomes in the community. This task is challenging due to the complexity of microbiome composition, limited availability of known reference genomes, and usually insufficient sequencing coverage.

**Results:**

As an initial step toward understanding the complete composition of a metagenomic sample, we studied the problem of estimating the total length of all distinct component genomes in a metagenomic sample. We showed that this problem can be solved by estimating the total number of distinct k-mers in all the metagenomic sequencing data. We proposed a method for this estimation based on the sequencing coverage distribution of observed k-mers, and introduced a k-mer redundancy index (*KRI*) to fill in the gap between the count of distinct k-mers and the total genome length. We showed the effectiveness of the proposed method on a set of carefully designed simulation data corresponding to multiple situations of true metagenomic data. Results on real data indicate that the uncaptured genomic information can vary dramatically across metagenomic samples, with the potential to mislead downstream analyses.

**Conclusions:**

We proposed the question of how long the total genome length of all different species in a microbial community is and introduced a method to answer it.

**Electronic supplementary material:**

The online version of this article (10.1186/s12864-019-5467-x) contains supplementary material, which is available to authorized users.

## Background

It is now widely known that microbiomes or the ecological community of microbes living at a certain site of the human host such as the gut can play important roles in human health [1–5]. Metagenomic sequencing is a powerful technology for studying the microbiome by sequencing DNAs from all the genomes of its component microbes [5]. Since it is impossible to capture the full components of a microbiome, a ‘metagenomic sample’ is actually a subset of the target metagenome captured with the sequencing process, as a sample from a population in statistics [6]. The basic task of a metagenomic study is to read out the underlying information about the microbiome from the metagenomic sample.

For any genomic sequencing study, a fundamental property we need to consider is the sequencing coverage, which is the fraction of genomic materials that has been captured and sequenced. This, however, has been largely ignored in metagenomic studies [6]. The level of coverage of a metagenomic sample is of key importance for recovering the information about the microbiome. Variations caused by coverage differences between metagenomic samples can be wrongly attributed to biological reasons, resulting in misleading conclusions [6].

The question of estimating the coverage of a sequencing sample has been attracting researchers’ attention since the beginning of human genome project. In 1988, Eric S. Lander and Michael S. Waterman introduced the famous Lander-Waterman theory to show how well a genome can be recovered for a certain sequencing strategy [7]. It had played a key role in guiding the design and completion of the human genome project. Lander-Waterman theory was specially designed for single genomic sequencing projects. It is no longer suitable for most metagenomic data since the relative abundances of component genomes in a microbiome are very uneven and therefore the sequencing procedure violates the uniform distribution assumption [8]. This is also true for other types of sequencing projects like RNA-sequencing or ChIP-seq where distributions of components to be sequenced are uneven. Methods were therefore introduced to estimate the coverage or solve similar problems in such situations [8–12]. For example, Hooper et al. proposed a method to estimate the total number of genomic bins in a metagenome by assuming certain abundance distribution of the microbial composition [8]. Rodriguez et al. assessed the abundance-weighted coverage of a metagenomic sample by examining the redundancy among individual reads [10]. Daley and Smith introduced an empirical Bayesian method to predict the number of previously un-sequenced molecules that would be observed if additional reads were provided [9]. This method has been demonstrated powerful in different kinds of sequencing data such as ChIP-seq data and RNA-seq data, but its effectiveness on metagenomic data has not been studied.

For the genomic sequences that have been captured in a metagenomic sample, the basic information we want to get is what types of microbes are there at what abundances. This is referred to as taxonomy profiling. A straightforward way of taxonomy profiling is to map sequencing reads to reference genomes in known databases. Known microbial genomes only represent a small proportion of existing microbes. Even for the type of well-studied communities like human gut, it’s typical that around 30%–60% of sequencing reads in a metagenomic sample could not be mapped to any known microbial genomes [13]. Furthermore, it has been observed that the fraction of unmapped reads can vary dramatically across different samples in the same study, say, ranging surprising from 2 to 96% [14]. This type of between-samples variation is lost when relative abundances are calculated based on mapped reads. Ignoring such loss of information can be misleading in downstream analyses [5].

Mainly because of the incomplete coverage and the existence of unmapped reads, the genomes that can be profiled from a metagenomic sample are only a part of all genomes that exist in the microbiome. It is therefore desirable to make estimations on the genomes that have been missed. Even if it is not possible to make accurate estimations on the number of missed genomes and their relative abundances, any educated guess about any properties of missed genomes can provide useful information for the comparison of samples based on known genomes. In this paper, we study the problem of estimating the total length of all distinct genomes in a metagenomic sample. If we can estimate this with reasonable accuracy, we will know a lot about the missed genomes by subtracting those known and mapped genomes from the total. This is the same question as estimating the actual coverage of the unknown targeting whole microbiome by the observed sequencing reads in the metagenomic sample. In preparation of this manuscript, a similar question has been studied in [15], but the method requires both long reads and short reads. For most cases where only short reads are available, we found that this question can be solved by solving the related question of estimating the number of distinct k-mers in the metagenome if we have infinite sequencing depth. A statistical model is introduced to predict the number of distinct k-mers in a metagenome that have not been included in the observed data. And we define a k-mer redundancy index (*KRI*) that helps to estimate the total genome length from total distinct k-mer count. Since the underlying truth is unknown in any real metagenomic data, we simulated a set of synthetic metagenomic datasets for different situations of microbial composition. Experiments on these data showed that the proposed method works well.

## Methods

### Problem statements

The problem we study is to estimate the total length of distinct genomes in a microbiome based on the metagenomic sequencing data. A more accurate statement of this problem in practice depends on the criteria for two genomes to be identified as distinct from each other. This is a complicated taxonomic question considering the wide existence of strains and sub-strains within each microbial species. To focus on the key mathematic problem behind the question, we simply assume that genomes from the same species are same while genomes from different species are distinct. We will give further discussion about this later in the “Estimating *KRI* of the distinct genome set” section.

### Understanding DNA sequence as a collection of k-mers

**Fig. 1:**
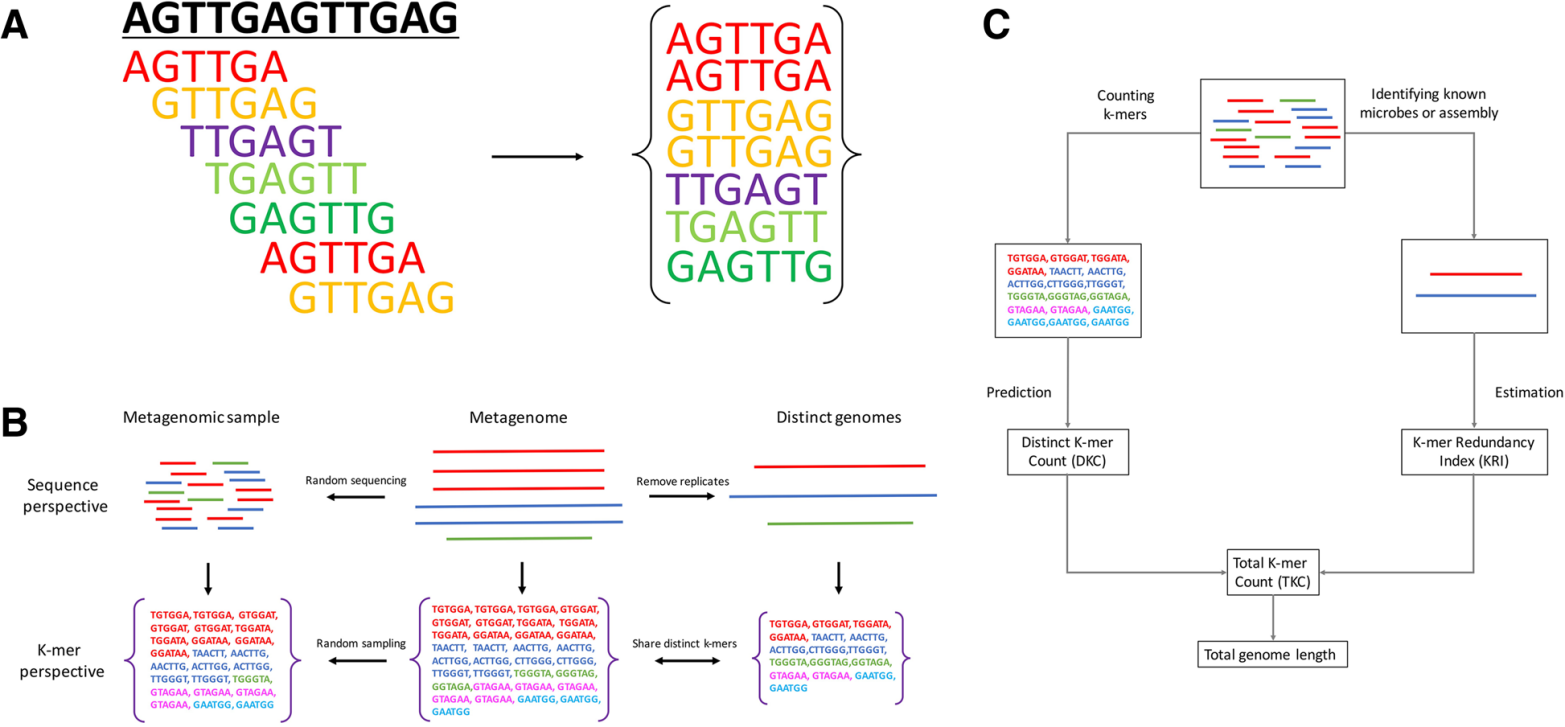
Overview of the proposed method. **a** An illustration of understanding DNA sequence as a collection of k-mers. In this simple case, sequence length *L*=12, *k*=6 for the k-mer counting, *TKC*= *L*−*k*+1=7, *DKC*=5, *KRI*=*TKC*/*DKC*=1.2. **b** Relationships between metagenome, metagenomic sample and the set of distinct genomes in the metagenome. **c** Workflow of the proposed method

A DNA sequence can be viewed as a collection of k-mers by breaking the sequence into nucleotide substrings of length k, as illustrated in Fig. 1a. From the k-mer perspective, we define total k-mer count (*TKC*), distinct k-mer count (*DKC*) and k-mer redundancy index (*KRI*) as three properties of a sequence. *TKC* is the number of all k-mers obtained when breaking a sequence into k-mers. *DKC* is the amount of distinct k-mers, i.e., the amount of remaining k-mers after removing all replicates of k-mers. *KRI* is defined as the ratio of *TKC* and *DKC*, which reflects the degree of repetition of k-mers in the sequence. The values of these three properties depend on the target sequence and the selection of k-mer size (*k*). For a given *k*, any of the three properties can be obtained if the other two are provided. For example, *TKC*=*DKC*∗*KRI*, which means *TKC* is achievable if we know *DKC* and *KRI* of a k-mer collection. Obviously, for a sequence of length *L*, *TKC*=*L*−*k*+1, indicating that *TKC* can be roughly taken as the sequence length if *L*≫*k*, which is satisfied when studying genomes using small k-mers. These simple mathematical relations form the basic idea of our work.

Similarly, a set of sequences can also be treated as a collection of k-mers by breaking every single sequence into k-mers. Therefore, a metagenomic sample, the metagenome and the set of distinct genomes in a metagenome can all be viewed as a collection of k-mers, respectively, as illustrated in Fig. 1b.

### Overview of our solution

From the k-mer perspective, our aim of estimating total genome length of all distinct genomes in a metagenome is equivalent to estimating *TKC* of the set of distinct genomes (Fig. 1b). Since it is impossible to count *TKC* of the true metagenome from the metagenomic sample due to finite sequencing coverage and unknown genome composition, we predict *TKC* of the distinct genome set by estimating its *DKC* and *KRI* separately (Fig. 1c). A metagenome and the corresponding set of distinct genomes of all its components differ only in genome abundances, they share the same distinct k-mers and have equal *DKC*s. We estimate *DKC* of the metagenome from the observed metagenomic data by modeling the sequencing event as a Poisson sampling procedure. *KRI* of the distinct genome set can be estimated based on known genomes detected in the metagenomic sample. Finally, the total genome length, which is roughly equal to *TKC*, can be achieved simply by taking the product of *KRI* and *DKC*.

### Predicting *DKC* of the metagenome

A metagenomic sample can be viewed as a subset of the metagenome obtained by random sampling, as illustrated in Fig. 1b. *DKC* of a metagenomic sample can be readily obtained by counting k-mers in the sequences, either from the original sequencing reads or from the assembled scaffolds. We need to estimate the number of k-mers in the metagenome that have not been covered in the metagenomic sample. The frequency that a given k-mer *i* is sequenced, denoted as *x*_*i*_, can be modeled as a Poisson distribution with an unknown parameter *λ*_*i*_. The probability that k-mer *i* will not been sequenced is $e^{-\lambda _{i}}\phantom {\dot {i}\!}$. We call these k-mers as uncaptured k-mers. Although the frequencies of k-mers overlapping with each other are dependent, such limited dependence can be well-approximated by assuming independence [16, 17]. Therefore, we further assume that *λ*_*i*_ independently and identically follow some unknown distribution *μ*(*λ*), the number of uncaptured k-mers is

1 $$\begin{array}{@{}rcl@{}} N\int \limits_{0}^{\infty} e^{-\lambda} \mathrm d\mu(\lambda) \end{array} $$

where *N* is the *DKC* of the metagenome. Since both *N* and *μ*(*λ*) are unknown, we are not able to calculate the value of (1) directly. Fortunately, the frequencies of captured k-mers in the metagenomic sample also contain information about *N* and *μ*(*λ*), which would help us to estimate the value of (1). Let *n*_*j*_ denote the number of k-mers that appear *j* times in the metagenomic sample. The expectation of *n*_*j*_ can be written as

2 $$\begin{array}{@{}rcl@{}} E(n_{j}) = N\int \limits_{0}^{\infty} e^{-\lambda}\lambda^{j}/j! \mathrm d\mu(\lambda) \end{array} $$

If we take the observation *n*_*j*_ as its expectation *E*(*n*_*j*_), the mathematical problem of estimating the number of uncaptured k-mers can be formulated as:

**Given observations** *n*_1_,*n*_2_,*n*_3_,…,*n*_*M*_, **which follow the formula**

$$ {n_{j}= N \int \limits_{0}^{\infty} e^{-\lambda}\lambda^{j}/j! \mathrm{d} \mu(\lambda)} $$

**where** *N***and** *μ*(*λ*)**are unknown. Find the value of**

$${N\int \limits_{0}^{\infty} e^{-\lambda}\mathrm d\mu(\lambda)} $$

To solve this mathematical problem, let *ω*(*λ*)=*Nλe*^−*λ*^, *m*_*i*_=(*i*+1)!*n*_*i*+1_, the problem can be re-written as

***Given observations*** *m*_0_,*m*_1_,*m*_2_,…,*m*_*M*−1_, ***which follow the formula***

$${m_{j}= \int \limits_{0}^{\infty} \lambda^{j}\omega(\lambda)\mathrm{d}\mu(\lambda)} $$

***where*** *ω*(*λ*)***and*** *μ*(*λ*)***are unknown. Find the value of***

$${\int \limits_{0}^{\infty} \frac{1}{\lambda} \omega(\lambda) \mathrm d\mu(\lambda)} $$

This is a special type of Gaussian quadrature problem that can be solved using the Golub-Welsch algorithm [9, 18]. The final estimation of (1) can be written as

3 $$\begin{array}{@{}rcl@{}} N\int \limits_{0}^{\infty} e^{-\lambda}\mathrm{d}\mu(\lambda) \approx \sum_{i=1}^{M} \frac{\alpha_{i}}{\lambda_{i}} \end{array} $$

where *α*_*i*_ and *λ*_*i*_ are decided by the Golub-Welsch algorithm taking *m*_0_,*m*_1_,*m*_2_,…,*m*_*M*−1_ as the input. *DKC* of the metagenome is finally achieved by adding this estimated uncaptured number of k-mers to *DKC* of the metagenomic sample. The variability and reliability of the estimation can be reflected by the confidence interval achieved by the bootstrap method.

### Estimating *KRI* of the distinct genome set

To precisely estimate *KRI* of the set of distinct genomes of a metagenome, one needs to know all different genomes in the metagenome, which is usually unachievable due the existence of many unknown microbes. To deal with this problem, we reasoned that *KRI* of a genome set can be well estimated use only part of the genomes in it. Therefore, we can use known genomes detected in a metagenomic sample to estimate the *KRI* of the whole distinct genome set. In practice, we first apply MetaPhlan2 [19] and GOTTCHA [20] on the metagenomic data to identify known species in the metagenome. For each detected species, we select one of its reference genomes from the database [9] to form a genome set. An alternative way to form the genome set is to take the assembled scaffolds as detected genomes. We estimated the *KRI* of this set of detected genomes as the *KRI* of the distinct genome set.

The way of selecting known genomes to estimate *KRI* actually decides the criteria of identifying distinct genomes in our work. Since we select only one genome for each detected species to estimate the *KRI* of the set of distinct genomes, the estimation is restricted to species level, even if two strains of the same species were detected in the metagenomic sample. If we include genomes for all detected strains in the *KRI* estimation, the estimation will be at strain level.

### Implementation of the method

We first adopt Pollux [21] to correct the sequencing error in the metagenomic samples. Counting all k-mers in a metagenomic sample can be computationally heavy. We employ jellyfish2 [22], one of the fastest k-mer counting approaches, for the k-mer counting step. We use the Golub-Welsch algorithm implemented in preseq [9, 17] to estimate the distinct k-mer count. MetaPlan2 [19] and GOTTCHA [20] are used to identify the known species from the metagenomic sample. Genomes for those known species are selected from existing database [23] to estimate the *KRI* for the whole community.

### Simulated metagenomic datasets

Due to the complexity of real-world microbiome compositions, it is hard, if possible, to find real metagenomic data that have complete true answer of all components. To test the performance of our method, we simulated several microbial communities of different situations and generate synthetic metagenomic samples. We simulated communities with 10 species and 50 species as representatives of a simple case and a more complicated case. We used three types of composition abundance distributions to form microbial communities of low, medium and high complexities (LC, MC and HC) following the way of a previous simulation study [24]. LC, MC and HC are defined based on the number of dominant microbe who has a high relative abundance. LC has only one dominant microbe. MC has two or more dominant species. HC has no dominant species. The fraction of information captured by the metagenomic data is of key importance for estimating the total genome length. To reflect this property of a metagenomic sample, we define initial coverage as the fraction of distinct k-mers in the set of distinct genomes of the target community included in the sequencing data. For each community, metagenomic samples of different reads numbers were generated to simulate the situation of different sequencing depths and the initial coverages of the community. To check how robust the method is to random effect, we use three random seeds to generate samples for the same parameters. In total, 225 metagenomic samples with 10 species and 243 samples with 50 species were generated with an in-house simulation tool [25]. Beside the error-free samples, we also generated a set of metagenomic samples with sequencing errors for each community.

We did some simple simulations to show that *KRI* of a genome set can be estimated using part of all genomes. We simulated four metagenomes with 10, 50, 100 and 200 species, respectively. For each metagenome, we randomly select 60% of its component genomes as known ones to estimate the *KRI* of the whole metagenome. Although in real world, the known microbes are not randomly selected from the nature, the order in which they were known has nothing to do with their sequence contents. Therefore, we believe such random selection is reasonable.

### Real metagenomic datasets

We select two datasets to conduct our method on. One dataset contains 65 oral metagenomic samples from Human Microbiome Project (HMP) [26] and the other consists of 145 human gut metagnomic samples, including 71 from normal people and 74 from type 2 diabetes patients [27].

## Results

### Results on simulated metagenomic datasets

**Fig. 2:**
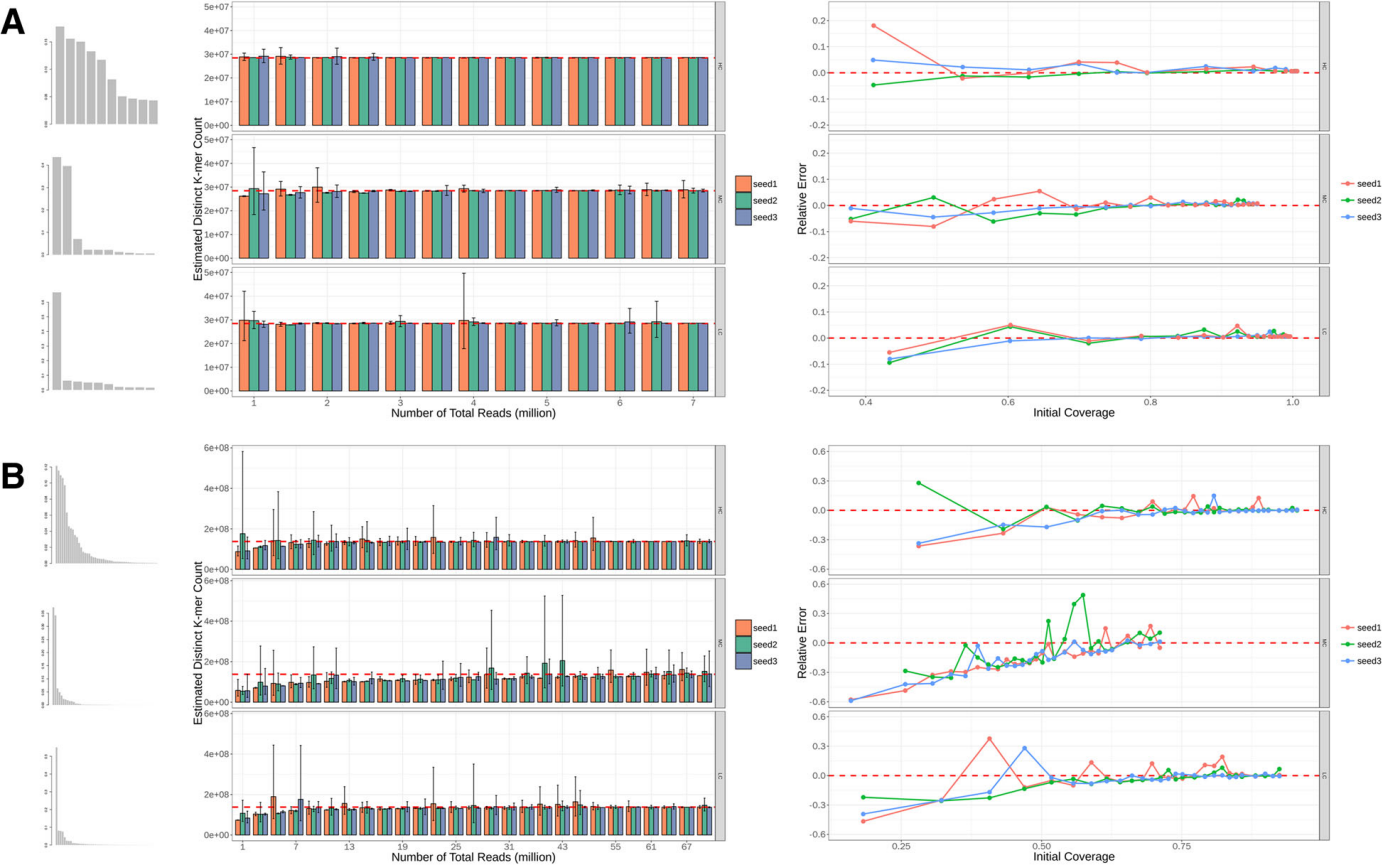
Different microbial communities are simulated to test the performance of the proposed method. (**a**) Results for microbial communities with 10 species. The three histograms on the left show the abundance distributions of different simulated communities. The middle panel shows the estimation results of distinct k-mer count. Each bar represents an estimation result based on a synthetic metagenomic sample and the error bar shows the 95% bootstrap confidence interval of the estimation. The black dash line is the true distinct k-mer count. The right panel shows how the relative error goes as the initial coverage increases (*k* = 20). (**b**) The same as (**a**) except that the species number is 50. (Note that some of the samples with 10 species are not shown in the barplot, see Additional file 1: Figure S1 for all samples with 10 species)

We tested our method on all synthetic metagenomic samples. Fig. 2 shows how well the number of distinct k-mers (*DKC*) in a community can be estimated from a metagenomic sample. The whole figure contains two parts, showing results for communities with 10 species and 50 species, respectively. Each part consists of three panels, displayed from left to right. Further explanations about each panel are given in the figure caption. As expected, the overall prediction in samples with 10 species is better than in samples with 50 species. Communities with high complexity achieve best prediction accuracy among those three kinds of abundance distributions. This agrees with the intuition that the more even the abundance distribution is, the better the prediction will be. The performances on communities with medium complexity are the worst. This is because the two dominant species make up more than 70% of the community, which means that most of the reads are sequenced from them. Since less than 30% of the reads come from the rest of all species, only a small part of information about their genomes is reflected in the sequencing data, leading to the bad performance, especially when sequencing depth is low. We also show how the performance goes when the initial coverage increases. The performance is measured by relative error, defined as the difference between estimated value and the true value divided by the true value. In general, the performance gets better as the initial coverage increases. Another interesting observation is that, for most cases, Golub-Welsch algorithm gives a good estimation which trends to be no larger than the ground truth, and the corresponding bootstrap confidence interval is usually small. For the exaggerated estimations, Golub-Welsch algorithm is more likely to give a large bootstrap confidence interval. Therefore, Golub-Welsch algorithm provides a reliable estimation of the lower bound of DKC, as suggested in preseq [9].

### Effects of K and sequencing errors

**Fig. 3:**
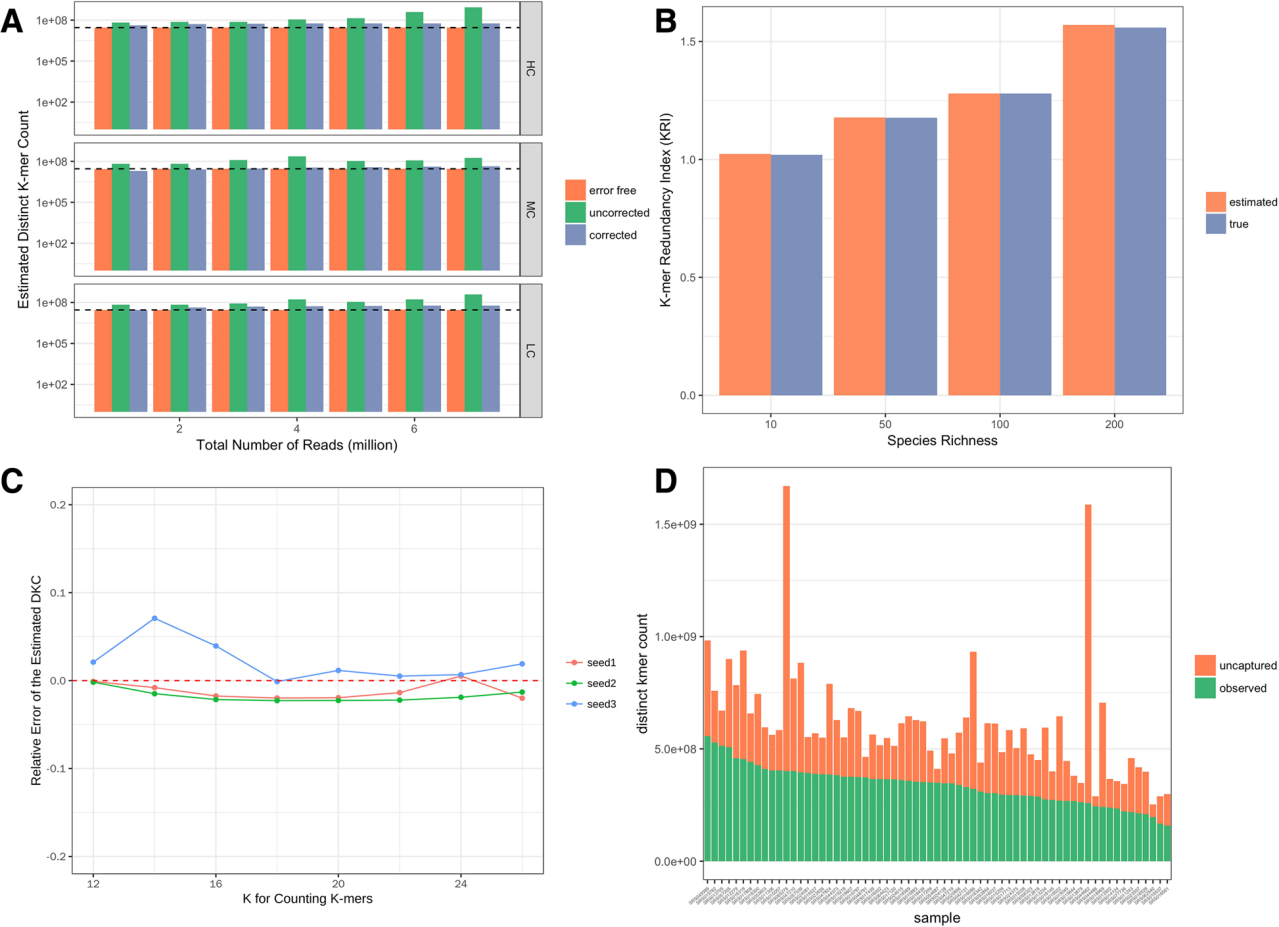
**a** Performance on metagenomic data with sequencing errors. **b** True and estimated K-mer Redundant Index (KRI) in different metagenomics communities. About 60% of the species are randomly chosen as the known species to estimate the KRI of all species. **c** Results of different selections of K. Simulated metagenomic sample with 50 speices and high complexity of the abundance distribution was used. **d** Results on HMP Tongue Dorsum datasets

To see how the parameter k affects the results, We chose different k to do the estimation for a simulated metagenomic sample (50 species, high complexity, 25 million reads). Results show that the estimation is robust to the selection of k (Fig. 3c).

Despite the good performance on error-free sequencing data, the Golub-Welsch algorithm can given bad prediction when the sequencing data contains errors (Fig. 3a). Sequencing errors introduce novel k-mers that should not exist in the data. A higher fraction of low-count k-mers will be considered by the algorithm as the implication of more low-abundant microbes. Therefore, sequencing errors lead to exaggerated estimation of total distinct k-mers and this exaggeration grows as the sequencing depths increases (Fig. 3a, green bars). To solve this problem, we use Pollux [21] to correct the sequencing error before counting k-mers. Results on simulation data show that the performance can be under control after correcting the sequencing errors (Fig. 3a, blue bars).

### Comparison between different methods

Besides Golub-Welsch algorithm, we also applied the major algorithm rational function approximation (RFA) in preseq on the simulated metagenomic samples with 50 species (Additional file 1: Figure S2) and compared its performance with Golub-Welsch algorithm. Both methods achieve a good performance and each present their own strength (Additional file 1: Figure S3). RFA outperforms Golub-Welsch algorithm in the median complexity communities (two species with a total relative abundance higher than 70%), indicating a stronger ability of extrapolation. For communities with high complexity or low complexity, Golub-Welsch algorithm makes stable and accurate results with only few exceptions. RFA also gives a good result, but with a slight trend to exaggerate the estimation.

### Estimating KRI using known species

There’s a gap between distinct k-mer count (*DKC*) and total genome length or *TKC*. We use *KRI* to bridge this gap as introduced above. For simulated metagenomic samples, GOTTCHA succesfully identified most species therefore led to a perfect estimation of KRI. We did some simple simulations to show that *KRI* of a genome set can be estimated using part of all genomes. In general, *KRI* of the community increases as there are more species in the community, as shown in Fig. 3b. The result shows that *KRI* of a community can be well estimated use only part of the species, which demonstrates the feasibility of estimating *KRI* of a community based only on known species.

### Results on real metagenomic datasets

**Fig. 4:**
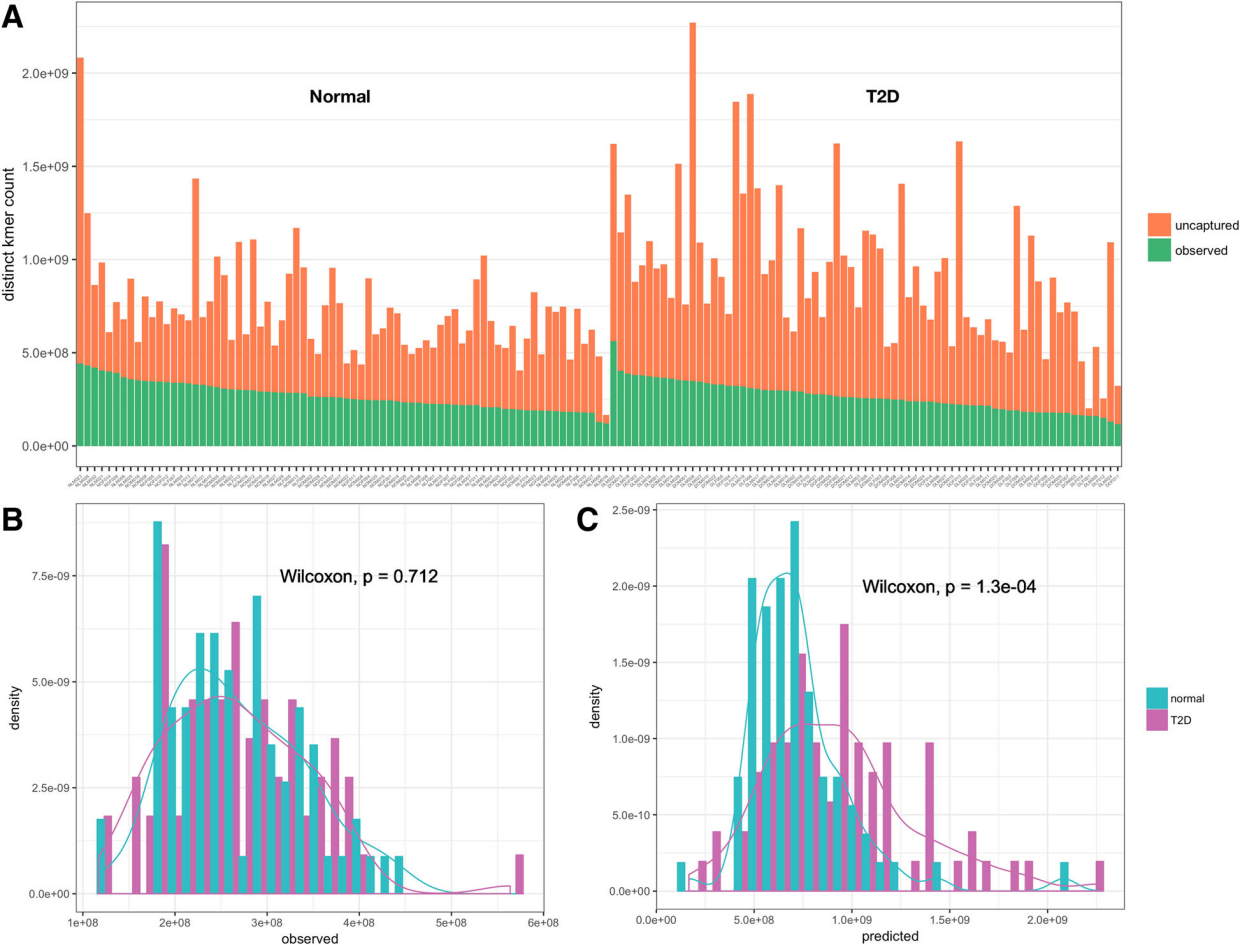
Results on T2D metagenomic datasets. **a** Observed and estimated k-mer count. **b** Histogram and density of the observed distinct k-mer count. **c** Histogram and density of the predicted distinct k-mer count

We applied our method on the two selected datasets (Figs. 3d and 4). One general observation in the results is that, the number of uncaptured k-mers can differ a lot between samples, even when the observed k-mer counts are similar (Figs. 3d and 4a). Further comparison between normal samples and T2D samples shows that the predicted distinct k-mer counts present significant difference while observed k-mer counts do not (Fig. 4c and d). In the original study, it was reported that the difference of within-sample diversity (entropy of gene abundance) between normal group and T2D group is not significant [27]. Since the gene abundances were calculated based only on extracted sequence data, chances are that the significance had been masked by ignoring the difference in the ’unseen’ information.

## Conclusion and discussion

In this paper, we proposed the question of ‘how long the total genome length of all different species in a microbial community is’ and introduced a method to answer it. This is an important step toward the estimation of unknown and unseen component genomes in a microbiome. We invented a k-mer-based strategy to liberate the reliance on the limited microbial reference genomes so that unknown species can be included in the estimation. To explore the information that has not been directly captured in the metagenomic sample, we developed a statistical method to estimate the number of uncaptured k-mers. Distinct k-mer count was multiplied by the k-mer redundancy index (*KRI*), an index defined to reflect the repetition of k-mers and estimated from known species, to get the total genome length. Performance on the simulation data shows that the proposed method works well, and the precision of the estimation is mainly affected by factors including the sequencing error, the initial coverage of the community and the complexity of the microbial diversity.

Extracting information from the metagenomic data is the foundation of downstream analysis. The complex nature of microbial community and inadequate microbial diversity represented in existing databases make it challenging to extract the full information. A metagenomic sample can capture only part of the information about the microbial community due to its complexity, among which only part can be extracted due to the limited known references. Ignoring these ‘uncaptured’ and ‘unknown’ information can mislead downstream analyses. In the work of estimating total genome length, we adopted the reference-free strategy to include the ‘unknown’ information and a statistical model was employed to estimate the ‘uncaptured’ part so that the completeness of the extracted information can be pursued to the maximum. The experiments on simulated data showed the feasibility of the proposed method and results on real datasets revealed that downstream analyses may be biased if ’unseen’ information is ignored. Further studies are needed in the future to explore ways by which the estimated total metagenome length can help to better extracting information about unknown or uncaptured species from the metagenomic data and comparing metagenome samples.

## Additional file

Additional file 1 This file contains **Figure S1** – **Figure S3**. (PDFk 6194 kb)

## Abbreviations

DKC: Distinct k-mer count
KRI: K-mer redundancy index
TKC: Total k-mer count
