# Supplementary material for: Estimating the total genome length of a metagenomic sample using k-mers
Source: BMC Genomics. 2019 Apr 4;20(Suppl 2):183. doi: 10.1186/s12864-019-5467-x (PMC6456951; doi:10.1186/s12864-019-5467-x)
Supplement: Supplementary file 1 — This file contains Figure S1 – Figure S3. (PDFk 6194 kb) [file 12864_2019_5467_MOESM1_ESM.pdf]

Estimating the Total Genome Length of a Metagenomic Sample Using K-mers

(Supplementary Materials)

Kui Hua[1,2], Xuegong Zhang[1,2,3,*]
[1]MOE Key Laboratory of Bioinformatics Division and Center for Synthetic \& System Biology, BNRIST, Beijing 100084, China
[2]Department of Automation, Tsinghua University, Beijing 100084, China
[3]School of Life Sciences, Tsinghua University, Beijing 100084, China
[*] Corresponding author. E-mail: zhangxg@tsinghua.edu.cn.

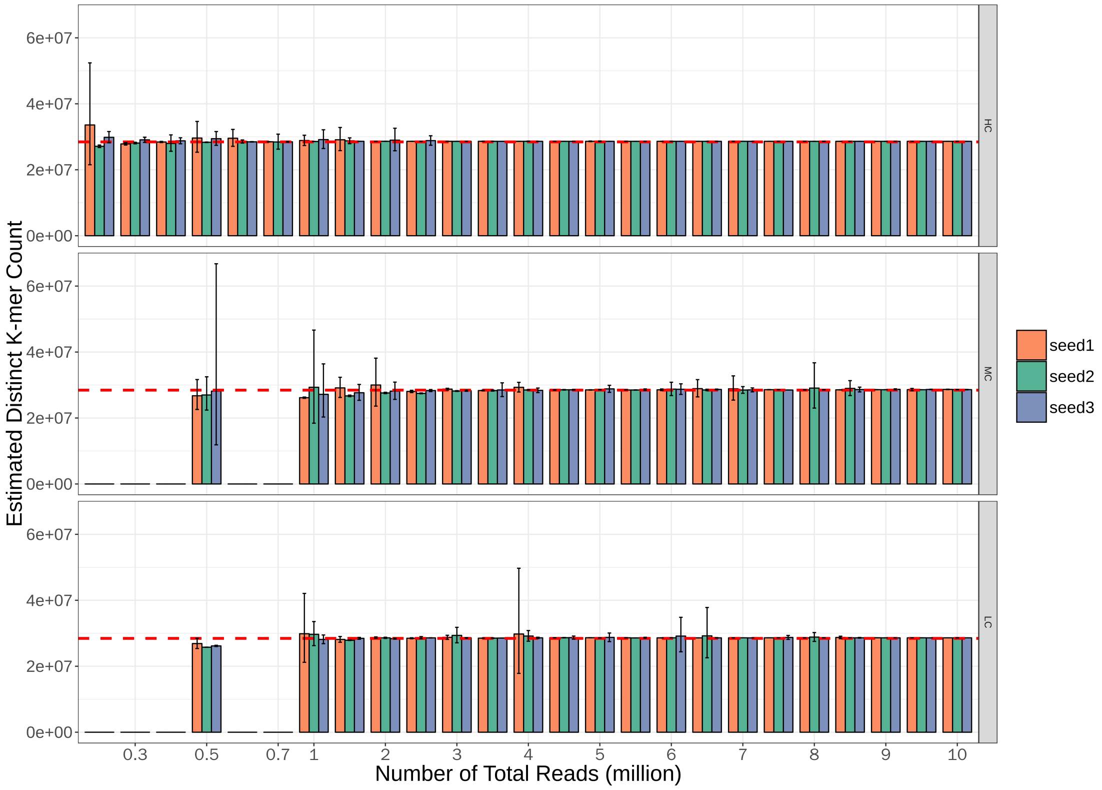

Figure S1. Results of all simulated metagenomic samples with 10 species. The abundance distribution of HC community is relative even compared to MC and LC communities. So the initial coverage can be high even with a small sample size. To see how the method works when the initial coverage is low, we specially simulated more samples with smaller sample size for HC community.

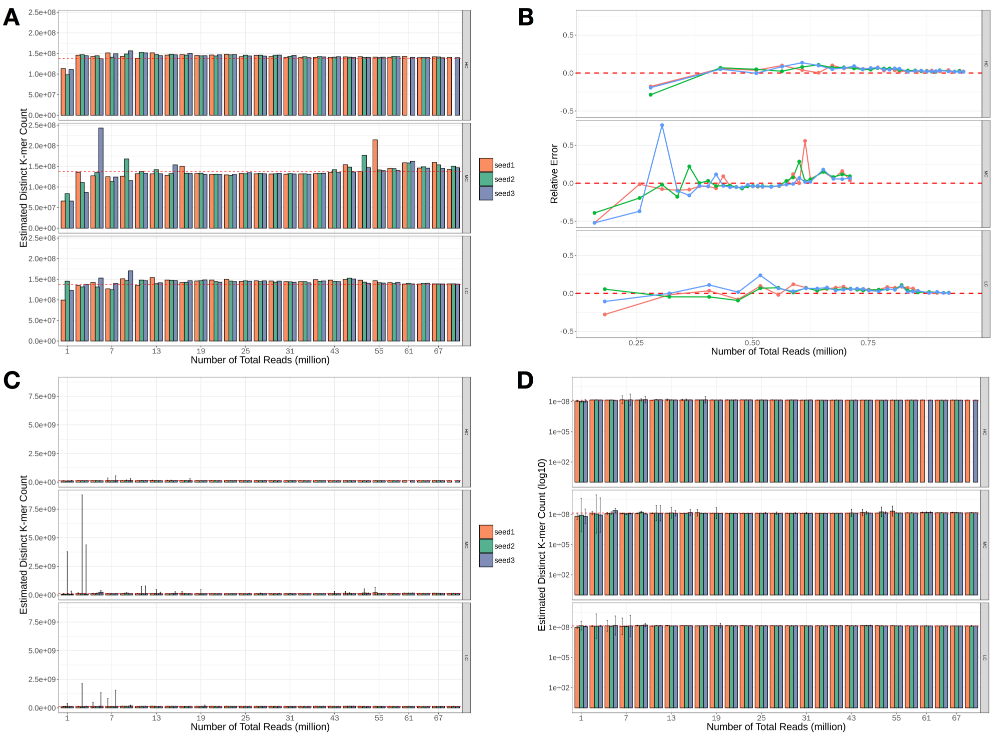

Figure S2. Performance of preseq (RFA). The meaning of the axis in the figure is the same as in figure 2.

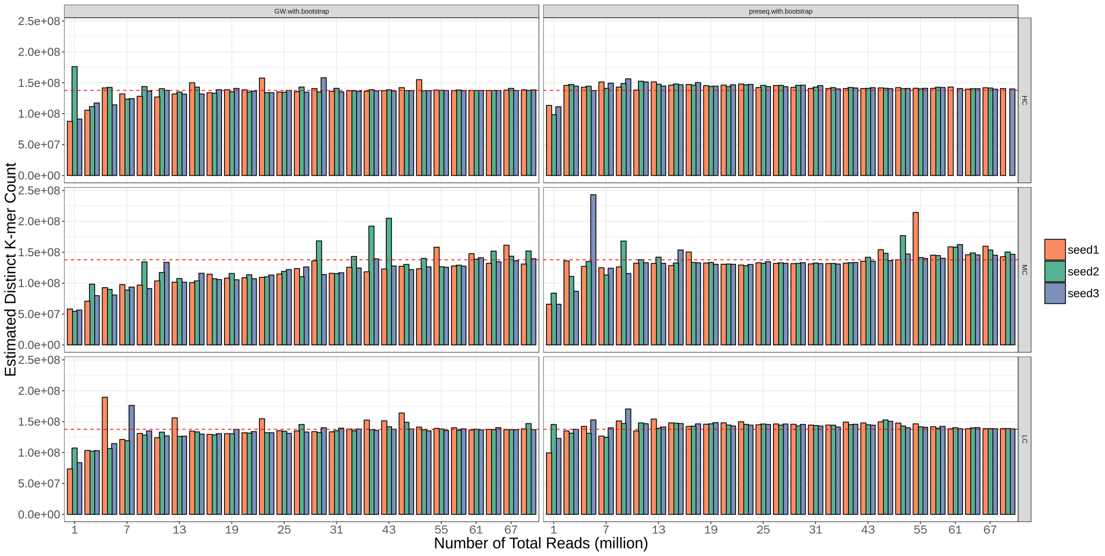

Figure S3. Comparison between Golub-Welsch algorithm (left panel) and RFA in preseq (right panel).
